# A next-generation sequencing-based strategy combining microsatellite instability and tumor mutation burden for comprehensive molecular diagnosis of advanced colorectal cancer

**DOI:** 10.1186/s12885-021-07942-1

**Published:** 2021-03-16

**Authors:** Jian Xiao, Wenyun Li, Yan Huang, Mengli Huang, Shanshan Li, Xiaohui Zhai, Jing Zhao, Chan Gao, Wenzhuan Xie, Hao Qin, Shangli Cai, Yuezong Bai, Ping Lan, Yifeng Zou

**Affiliations:** 1 Department of Medical Oncology, The Sixth Affiliated hospital of Sun Yat-sen University, Guangzhou, China; 2 Department of Public Health and Preventive Medicine, School of Medicine, Jinan University, Guangzhou, China; 3 Department of Pathology, The Sixth Affiliated Hospital, Sun Yat-sen University, Guangzhou, China; 4 The Medical Department, 3D Medicines Inc., Shanghai, China; 5 Research and Development Institute of Precision Medicine, 3D Medicines Inc., Shanghai, China; 6 Department of Colorectal Surgery, The Sixth Affiliated hospital of Sun Yat-sen University, 26 Yuancun Er Heng Road, Guangzhou, 510655 Guangdong China

**Keywords:** Microsatellite instability, Tumor mutation burden, Next generation sequencing, Colorectal cancer, Immune checkpoint inhibitor

## Abstract

**Background:**

Mismatch repair (MMR)/microsatellite instability (MSI) and tumor mutational burden (TMB) are independent biomarkers that complement each other for predicting immune checkpoint inhibitors (ICIs) efficacy. Here we aim to establish a strategy that integrates MSI and TMB determination for colorectal cancer (CRC) in one single assay.

**Methods:**

Surgical or biopsy specimens retrospectively collected from CRC patients were subjected to NGS analysis. Immunohistochemistry (IHC) and polymerase chain reaction (PCR) were also used to determine MMR/MSI for those having enough tissues. The NGS-MSI method was validated against IHC and PCR. The MSI-high (MSI-H) or microsatellite stable (MSS) groups were further stratified based on tumor mutational burden, followed by validation using the The Cancer Genome Atlas (TCGA) CRC dataset. Immune microenvironment was evaluated for each subgroup be profiling the expression of immune signatures.

**Results:**

Tissues from 430 CRC patients were analyzed using a 381-gene NGS panel. Alterations in *KRAS*, *NRAS*, *BRAF*, and *HER2* occurred at a significantly higher incidence among MSI-H tumors than in MSS patients (83.6% vs. 58.4%, *p* = 0.0003). A subset comprising 98 tumors were tested for MSI/MMR using all three techniques, where NGS proved to be 99.0 and 93.9% concordant with PCR and IHC, respectively. Four of the 7 IHC-PCR discordant cases had low TMB (1.1–8.1 muts/Mb) and were confirmed to have been misdiagnosed by IHC. Intriguingly, 4 of the 66 MSS tumors (as determined by NGS) were defined as TMB-high (TMB-H) using a cut-off of 29 mut/Mb. Likewise, 15 of the 456 MSS tumors in the TCGA CRC cohort were also TMB-H with a cut-off of 9 muts/Mb. Expression of immune signatures across subgroups (MSS-TMB-H, MSI-H-TMB-H, and MSS-TMB-L) confirmed that the microenvironment of the MSS-TMB-H tumors was similar to that of the MSI-H-TMB-H tumors, but significantly more immune-responsive than that of the MSS-TMB-L tumors, indicating that MSI combined with TMB may be more precise than MSI alone for immune microenvironment prediction.

**Conclusion:**

This study demonstrated that NGS panel-based method is both robust and tissue-efficient for comprehensive molecular diagnosis of CRC. It also underscores the importance of combining MSI and TMB information for discerning patients with different microenvironment.

**Supplementary Information:**

The online version contains supplementary material available at 10.1186/s12885-021-07942-1.

## Background

Over the past two decades, precision medicine has immensely transformed cancer management. Colorectal cancer (CRC) patients, in particular, significantly benefited from such progresses. The NCCN Guidelines for colon cancer have incorporated genetic tests such as *KRAS*, *NRAS*, *BRAF* and *HER2* to guide targeted therapy, microsatellite instability (MSI)/mismatch repair (MMR) to inform immune checkpoint inhibitor (ICI) treatment, and germline profiling of *APC*, *MMR*, *STK11*, *PTEN,* etc. to assess hereditary CRC predisposition [1, 2]. Ever since the Food and Drug Administration (FDA) approved pembrolizumab and nivolumab for treating MMR-deficient (dMMR)/MSI-high (MSI-H) advanced solid tumors and metastatic CRC, MMR and MSI, apart from their role as a hallmark of Lynch Syndrome (LS), have drawn widespread attention as predictive biomarkers to define the population most likely to benefit from ICI treatment [3–6].

Before massively parallel DNA sequencing became available, MMR and MSI detection mainly relied on immunohistochemistry (IHC) staining for the MMR proteins and polymerase chain reaction (PCR) evaluation of five highly conserved loci of the “Bethesda panel” respectively [7]. However, as a biopsy procedure can hardly produce sufficient tissue, especially in advanced cancers, for comprehensive profiling of MMR/MSI status along with an array of other genes of interest, a vast majority of patients are usually left with limited treatment options [8]. Therefore, there exists a demand for a strategy to simultaneously detect various biomarkers in one single assay.

As next-generation sequencing (NGS) emerges, multiple groups reported NGS panels detecting MMR/MSI as well as other clinically relevant biomarkers for CRC [9–12]. Despite their strong correlation with conventional methodologies, none of these studies attempted tumor mutational burden (TMB) estimation in parallel with MSI detection. Although an MSI-H phenotype is usually suggestive of a high TMB level, they do not always correlate. TMB, usually detected through panel-based NGS or whole exome sequencing, represents a measure of the number of mutations harbored by tumor cells. High TMB levels may often translate into high neoantigen loads, which in turn may stimulate the immune system to recognize and attack tumor cells, and thereby predict potential sensitivity to immunotherapy [13]. TMB has been demonstrated to be an independent biomarker in CRC that could further stratify MSI-H or microsatellite stable (MSS) subset for the likelihood of response to ICIs treatment, thereby maximizing clinical benefits while improving cost-efficiency [13, 14]. A strategy combining MSI and TMB determination is thus particularly appealing since it provides additional information for comprehensive molecular characterization of CRC patients.

In this work, we profiled the genetic landscape of Chinese CRC patients using a 381-gene NGS panel which integrated MSI and TMB calculation algorithms, followed by clinical validation of MSI diagnosis against IHC and PCR. TMB distribution among MSI-H and MSS subsets were also explored and the concordance between TMB and MSI statuses and the expression profiles of immune signature genes were analyzed using The Cancer Genome Atlas (TCGA) database.

## Methods

### Patients and study design

CRC patients were retrospectively included at the Department of Medical Oncology of The Sixth Affiliated Hospital of Sun Yat-sen University from June, 2016 to September, 2018. Patients were eligible if they had a histologically confirmed diagnosis of CRC and ≥ 20% tumor cell content in their tissue samples. Patients with multiple primary lesions were excluded. All formalin-fixed paraffin-embedded (FFPE) tissue samples obtained from surgery or biopsy were retrospectively analyzed using targeted NGS. For IHC and PCR testing, all of the patients identified as MSI-H by NGS were recruited, while MSS cases were randomly selected from the NGS-defined MSS patients. A single FFPE block was used for NGS, IHC, and PCR, and any sample with insufficient tissue for all three methods was excluded from the validation concordance analysis. The accuracy of MSI diagnosis by NGS was evaluated using both IHC and PCR as reference methods. The study was approved by the hospital’s ethics committee.

### The MasterView panel design and NGS

The MasterView panel was developed by 3DMed Inc., a College of American Pathologists (CAP)-accredited and Clinical Laboratory Improvement Amendments (CLIA)-certified laboratory, to detect 100 MSI loci, 4557 exons of 365 carcinogenic genes including *MMR* genes (*MLH1*, *MSH2*, *MSH6*, *PMS2*) and important CRC-related genes such as *KRAS, NRAS, BRAF, ERBB2,* and *POLE,* and 47 introns of 25 frequently rearranged genes. Detailed information on the 381 genes are provided in Supplemental Table S1.

A detailed experimental procedure was previously described [15]. Briefly, specimens < 1 mm in length or containing < 20% tumor cells were excluded from further analyses. 50–200 ng of DNA extracted from the FFPE tumor specimens were sheared into ~ 200 bp fragments, followed by sequencing on an Illumina Nextseq 550 with a PE75 read length. The average sequencing depth was 1000× for tumor samples and 300× for blood control samples. Nucleotide substitutions, indels, copy number variations and DNA arrangement were identified using an in-house validated pipeline as previously described [15]. All germline mutations were filtered out by comparison with adjacent normal tissues or blood controls. False-positive variants were filtered using an in-house developed script.

### MSI and TMB estimation using NGS

A Small Panel **N**ext-generation sequencing on MSI (SPANOM) algorithm was developed for MSI determination. SPANOM was designed in four steps. First, a list of 2539 microsatellite loci were initially selected and subsequently evaluated for coverage based on 145 samples (49 MSI-H and 96 MSS) with whole exome sequencing (WES), and the top 100 loci were included in the final panel. For each sample, the percentage of MSI loci that were defined as microsatellite unstable was calculated, and according to the international criteria for MSI test with more than 5 MSI loci [7], a percentage of above 0.4 was considered as MSI-H and otherwise MSS.

TMB was defined as the number of all somatic SNVs and indel variants per megabase of coding genome sequenced in Chinese CRC cohort. SNVs referred to synonymous & non-synonymous mutations, stop gain/loss, and splicing variants. Indels included both frameshift and non-frameshift insertions and deletions. Non-coding alterations were excluded from TMB calculation.

### MMR detection using IHC

FFPE samples were analyzed for the expression of MLH1, MSH2, MSH6 and PMS2 using the Envision method following the manufacturer’s instruction (ZSGB-Bio, China). All sections were microscopically scored by two pathologists in a blinded manner. Briefly, 10 high-power (× 400) view fields were randomly selected for each sample to score the expression of proteins of interest in tumor cells. Defects in MLH1, MSH2, MSH6 and PMS2 proteins (dMMR) were defined as complete absence of nuclear staining in tumor cells. Discordant cases between IHC and PCR were resolved by another independent CAP-certified lab.

### MSI determination using PCR

DNA was extracted from paired tumor and normal tissues. Multiplex PCR was conducted using the Microsatellite Instability Detection Kit (Microread, China), which allowed for the detection of six markers: BAT25, BAT26, NR-21, NR-24, NR-27, and Mono27 by co-amplification using 5 μl of DNA with a concentration of 3 ng/ml. The PCR program was as follows: denaturation at 95 °C for 5 min and 40 cycles of 95 °C for 30 s, 60 °C for 1 min and 70 °C for 1 min, followed by 15 °C for 40 min. The amplicons were subjected to capillary electrophoresis on an ABI 3500 DX Genetic Analyzer (Applied Biosystems), followed by analysis using the GeneMapper v4.1 software (Applied Biosystems). If the peak for a certain marker (referred to as a left shift or right shift) was present in a tumor sample but absent in the corresponding normal sample, that marker was considered instable. MSI status was classified into three categories: MSI-H, MSI-low (MSI-L), and MSS depending on the number of instable markers (≥2, ≥1, 0).

### Validation using the TCGA cohort

The WES, RNAseq and MSI (by PCR) data of 533 CRC patients were extracted from the TCGA database (https://portal.gdc.cancer.gov/). Experimental procedures regarding DNA and RNA extraction, library preparation, sequencing, quality control, and subsequent data processing were published previously by TCGA [16]. MSI status was evaluated by the TCGA consortium using a panel of four mononucleotide repeats (BAT25, BAT26, BAT40 and TGFBRII) and three dinucleotide repeats (D2S123, D5S346 and D17S250). TMB was defined as the total non-silent somatic mutation counts in coding regions in TCGA CRC cohort. The expression profiles of immune signatures such as immune activation genes, immune checkpoint genes were evaluated using RNAseq data [17–19].

### Statistical analyses

Statistical analyses and plotting were performed using the R software v3.6.0 (http://www.Rproject.org). Categorical variables were analyzed using the Pearson χ^2^ test and continuous variables were analyzed using a non-parametric Wilcoxon rank-sum test for unpaired samples. Multiple groups of normalized data were analyzed using non-parametric one-way ANOVA. RNA-seq data from TCGA were subjected to trimmed mean of M-values (TMM) normalization and voom transformation. ROC curve was used to determine the cut-off values for defining TMB-H versus TMB-L populstions. If not specified otherwise, all tests were two-tailed, and a *P*-value of < 0.05 was considered statistically significant. The P-value for multiple testing were adjusted using the false discovery rate (FDR).

## Results

### Genetic landscape of Chinese CRC patients

**Fig. 1:**
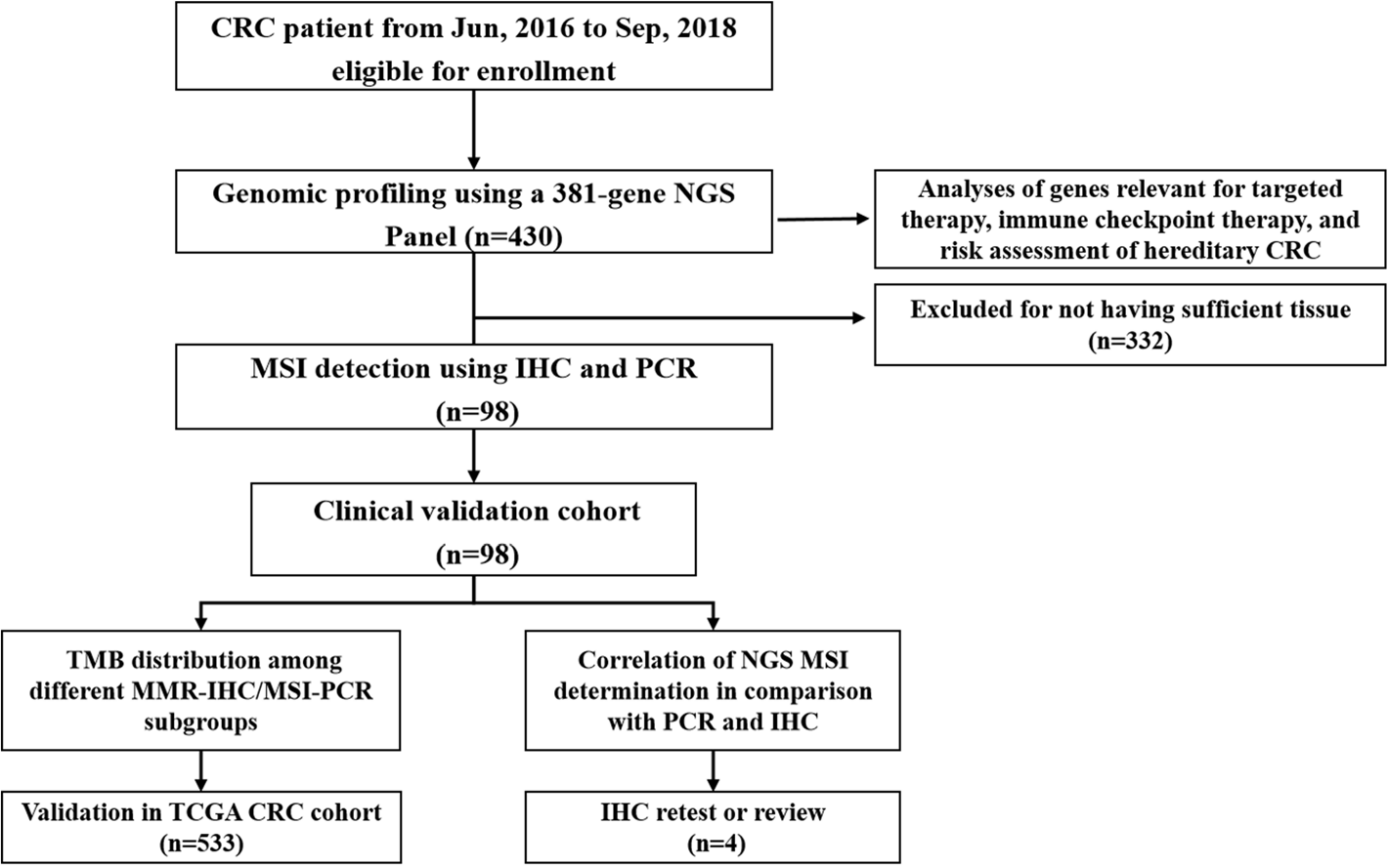
Patient Flow. Tissue samples were collected from 430 eligible CRC patients and subjected to genomic profiling using NGS. Of the 430 samples, 98 were also examined for MMR/MSI status using IHC and PCR. NGS MSI method was validated against IHC and PCR. The distribution of TMB between MSS and MSI-H tumors were also investigated using both the 98-patient cohort and the TCGA CRC cohort

**Fig. 2:**
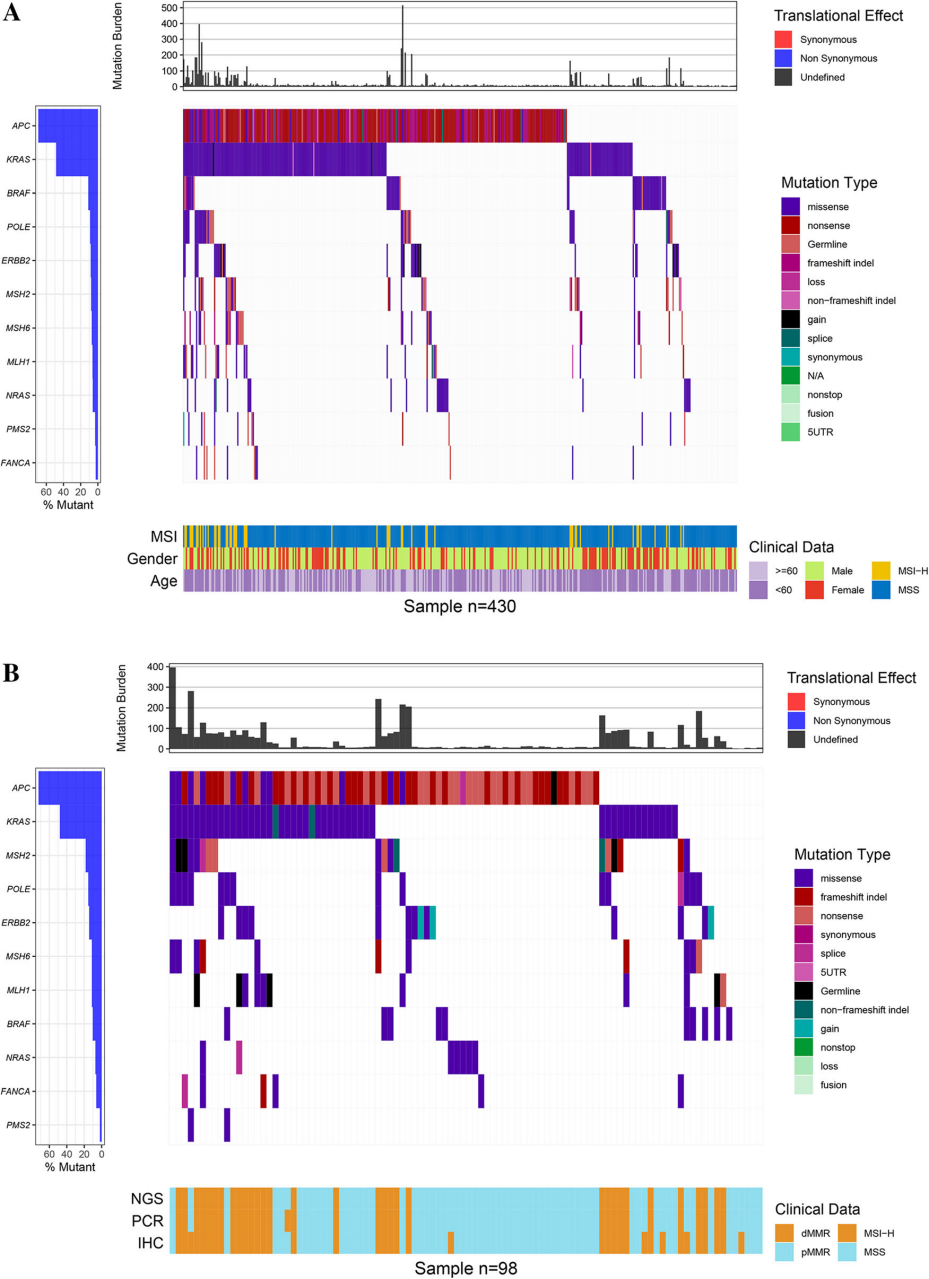
Genomic landscape of Chinese CRC patients using NGS. **a** Genomic landscape of 430 patients. The panel on the top shows the effects of the mutations at translational level while the panel at the bottom shows the MSI status, gender and age of each patient. The top 11 most frequently mutated genes are shown on the left. **b** Genomic landscape of the 98 tumors that were analyzed for MMR/MSI by IHC, PCR, and NGS. The bottom panel illustrates the MMR or MSI detection results for each patient

From June, 2016 until September, 2018, tissue samples from 430 patients with stage I-IV CRC were subjected to NGS analysis using the 381-gene MasterView panel (3DMed Inc.) (Fig. 1, Table S2). The average age of the patients was 55 years (interquartile range 18 to 84) and 65.1% of them (268/430) were male (Fig. 2a). The majority of the patients had advanced stage disease (30.5% with stage III and 54.4% stage with IV). In line with previous literature, the most frequently altered genes were *TP53* (77.6%, 334/430) and *APC* (69.7%, 300/430). MSI analysis revealed that among the 430 patients, 54 were MSI-H and 376 were MSS. When scrutinized for potentially actionable genetic alterations informing selection of or predicting response to targeted therapies, 45 MSI-H patients carried pathogenic or likely pathogenic mutations in *KRAS* (*n* = 37), *NRAS* (*n* = 1), *BRAF* (*n* = 7), and *HER2* (*n* = 3), while 220 of those diagnosed as MSS harbored alterations in *KRAS* (*n* = 170), *NRAS* (*n* = 18), *BRAF* (*n* = 27), and *HER2* (*n* = 14; 8 amplification, 5 point mutations, and 1 amplification & point mutation), indicating that significantly more MSS cases (41.4%, 156/376) than MSI-H patients (16.7%, 9/54) (*p* = 0.00079) were potentially sensitive to anti-EGFR monoclonal antibodies. Germline mutations were observed in 20 cases, of which 16 had defects in the *MMR* genes (8 *MLH1*, 7 *MSH2*, and 1 *MSH6*) that were indicative of LS and were all confirmed to be MSI-H according to NGS MSI analysis, 3 were altered in the *APC* gene (2 MSS and 1 MSI-H) and 1 harbored a mutation in the *FANCA* gene. Seven patients were found to carry pathogenic variations in *POLE* (6 MSS and 1 MSI-H) and as reported previously [20], the median TMB of the *POLE*-mutated patients was significantly higher than that of the total population (215.26 muts/Mb vs 8.13 muts/Mb, *p* < 0.001) (Supplemental Figure S1). Surprisingly, a tumor carrying a variation of unknown significance (VOUS) in *POLE* (p.L1327M) that has never been reported previously, also displayed a high TMB of 133.07 muts/Mb.

### Clinical validation of the MasterView panel-based NGS-MSI method in comparison with conventional PCR-MSI and IHC-MMR assays

**Fig. 3:**
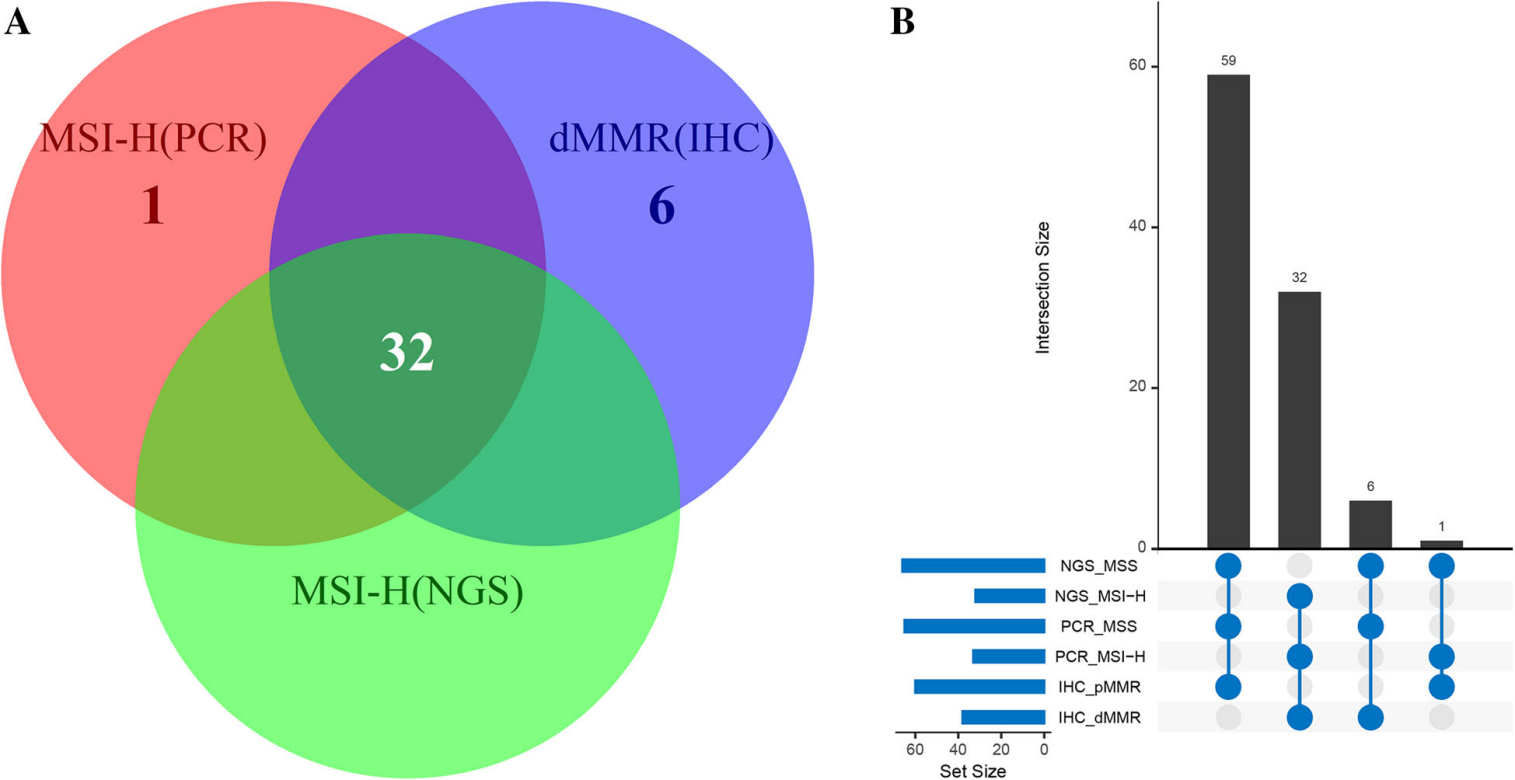
Concordance among IHC, PCR, and NGS for MMR/MSI detection. **a** A Venn diagram showing the overlap among the dMMR cases by IHC, MSI-H cases by PCR, and the MSI-H cases by NGS; **b** An Upset plot shows the detail of he consistent diagnosis result between NGS and conventional assays

Samples from 98 patients underwent investigation using NGS as well as conventional approaches IHC and PCR (Fig. 2b, Table S3). IHC detected deficient MMR protein expression in 38 patients (dMMR) while PCR identified 33 patients to be MSI-H. There was only one MSI- L case by PCR and it was categorized along with the MSS tumors in the subsequent analyses according to previous report [21]. The 32 cases overlapped between the two methodologies were confirmed to be MSI-H using NGS (Fig. 3a). Of the 60 MMR-proficient (pMMR) patients, one was MSI-H with PCR, but MSS with NGS, while the rest turned out to be MSS using both IHC and NGS (Fig. 3b). The NGS-MSI method demonstrated a 97.0% (32/33) sensitivity and a 100% (65/65) specificity compared to PCR, and an 84.2% (32/38) sensitivity and a 100% (60/60) specificity with respect to IHC (Table 1). The concordance rate was 99.0% between NGS and PCR, and 93.9% between NGS and IHC.

**Table 1:** MSI status using NGS-MSI against conventional PCR and IHC

PCR-MSI as Gold Standard			IHC-MMR as Gold Standard		
	PCR Positive	PCR Negative		IHC Positive	IHC Negative
NGS Positive	32	0	NGS Positive	32	0
NGS Negative	1	65	NGS Negative	6	60
Sensitivity = 97.0%(32/33)			Sensitivity = 84.2%(32/38)		
Specificity = 100%(65/65)			Specificity = 100%(60/60)		
Concordance Rate = 99.0%(97/98)			Concordance Rate = 93.9%(92/98)		

Intriguingly, there were 7 discordant cases between IHC and PCR, 6 dMMR-IHC/MSS-PCR and 1 pMMR-IHC/MSI-H-PCR (Table 2). The pMMR-IHC/MSI-H-PCR case was confirmed to be MSS with a TMB of 7.13 muts/Mb using NGS and therefore was possibly misdiagnosed by PCR. All of the 6 dMMR-IHC/MSS-PCR cases were also demonstrated to be MSS upon NGS testing, where 3 had enough tissue left and turned out to be pMMR upon repeat IHC by an independent CAP-certified laboratory and 1 was redefined as pMMR by reviewing the IHC slide, supporting the accuracy of our NGS method in case of discrepancy between PCR and IHC. The other 2 dMMR-IHC/MSS-PCR patients were found to harbor genetic alterations in the *MMR* genes in addition to defects in MMR protein expression. One patient had a truncation variant of the *PMS2* gene accompanied by the loss of PMS2 in IHC while the other carried a pathogenic *MLH1* somatic mutation R265C while testing negative for MLH1 and PMS2 proteins.

**Table 2:** Case analysis of seven patients with discordant results between IHC and PCR

Patient ID	Stage	Pathology	Tumor site	IHC interpretation	Absent protein of IHC	MSI-PCR	MSI-NGS	TMB (muts/Mb)	MMR relative alterations	Review or Retest of IHC
14,167	IV	Adenocarcinoma	Ascending colon	pMMR	None	MSI-H	MSS	7.1		Not enough tissue
31,713	IV	Adenocarcinoma	Rectum	dMMR	*MLH1* and *PMS2*	MSS	MSS	20.2	*MLH1* R265C and *BRAF* V600E	Not enough tissue
12,563	IV	Signet ring cell	Rectum	dMMR	*PMS2*	MSS	MSS	1.1		Retest as pMMR
11,640	IV	Adenocarcinoma	Ileocecal junction	dMMR	*PMS2*	MSS	MSS	7.1		Review as Misinterpretation
12,873	II	Adenocarcinoma	Ascending colon	dMMR	*PMS2*	MSS	MSS	280.9	*PMS2* p.R563* and *POLE* p.P286R	Not enough tissue
12,880	II	Adenocarcinoma	Rectum	dMMR	*MSH6*	MSS	MSS	3.2		Retest as pMMR
15,706	IV	Adenocarcinoma	Descending colon	dMMR	*MSH6*	MSS	MSS	8.1		Retest as pMMR

### TMB evaluation as a supplemental approach for CRC molecular diagnosis

**Fig. 4:**
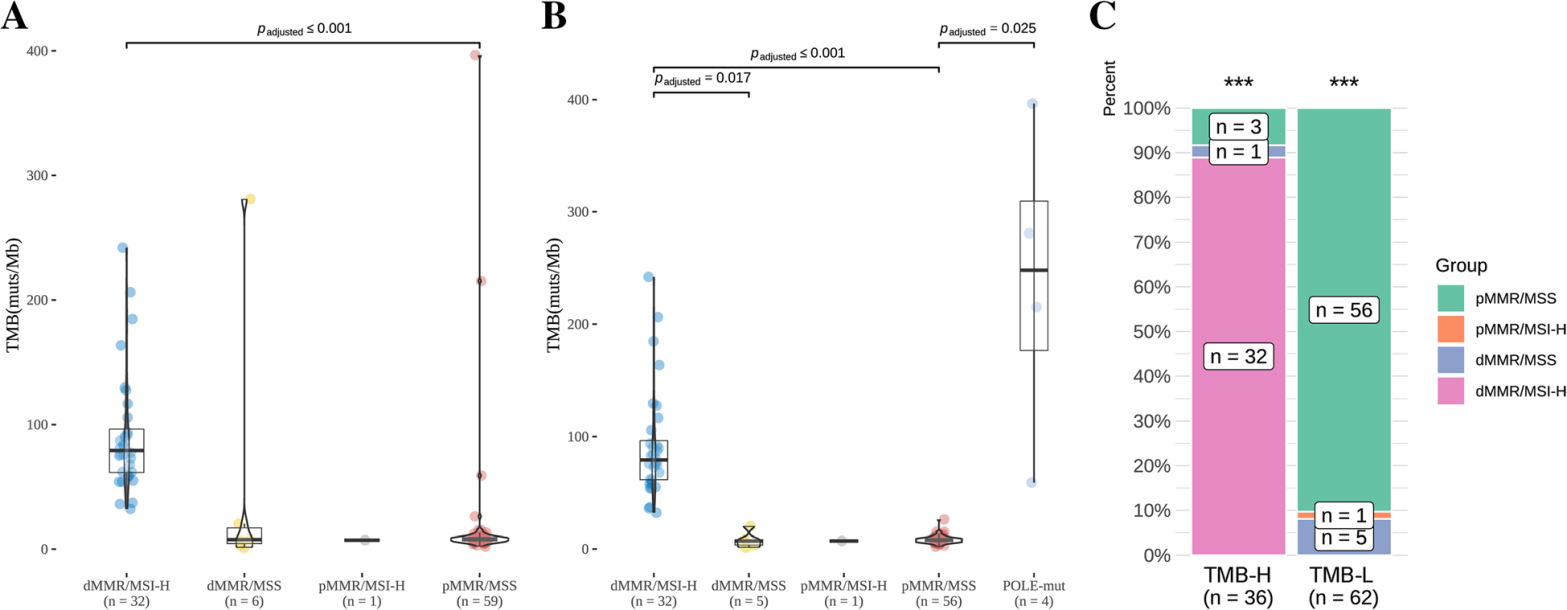
Correlation between TMB and MSI statuses. **a** TMB distribution across subgroups stratified according to MMR and MSI statuses determined using IHC and PCR; **b** TMB distribution across subgroups where POLE-mutated tumors were removed from their original groups and analyzed separately; **c**) Distribution of various MMR-IHC/MSI-PCR statuses between TMB-H versus TMB-L subsets as defined using a cut-off of 29 muts/MB

Due to the significance of TMB for predicting the efficacy of ICI treatment and previous reports that MSI and TMB statuses were not always consistent, we evaluated the TMB levels of different subsets stratified according to MMR and MSI statuses determined using IHC and PCR. As expected, the median TMB of the dMMR-IHC/MSI-H-PCR group was significantly higher than that of the pMMR-IHC/MSS-PCR patients (79.12 muts/Mb versus 8.13 muts/Mb, *p* ≤ 0.001), but not the discordant groups (Fig. 4a). Since *POLE*-mutated tumors tended to have higher TMB levels than their wild-type counterparts [22], they were removed from their original subgroups and analyzed separately. Similar to the pattern observed in the total population, the *POLE*-mutated group had a median TMB of 248.12 muts/Mb, significantly higher than that of the pMMR-IHC/MSS-PCR patients (8.13 muts/Mb) (*p* = 0.025), however, the differences between the *POLE*-mutated patients and the two discordant subgroups, the median TMB of which were both 7.14 muts/Mb, did not reach statistical significance, probably due to the small sample sizes (Fig. 4b). Following exclusion of the *POLE*-mutated cases, the median TMB of the dMMR-IHC/MSI-H-PCR subgroup became significantly higher than those of the pMMR-IHC/MSS-PCR group (79.12 muts/Mb vs. 8.13 muts/Mb, *p* ≤ 0.001) and the dMMR-IHC/MSS-PCR group (79.12 muts/Mb vs. 7.14 muts/Mb, *p* = 0.018).

Using a cut-off of 29 mut/MB to define high vs. low TMB, as trained using our clinical cohort and determined based on an optimal YOUDEN index of 0.9 with a sensitivity of 0.97 and a specificity of 0.94 (Supplementary Figure 2), the TMB-high (TMB-H) population encompassed the dMMR-IHC/MSI-H-PCR subset and 4 *POLE*-mutated patients, who were found to be MSS using both PCR and NGS (Fig. 4c). They were likely to respond to immunotherapy since *POLE* variations are often associated with sensitivity to ICIs, presumably secondary to high TMB levels (58.9 ~ 396.5 mut/MB), but could have been deprived of the opportunity to receive immunotherapy by just relying on MSI status. On the other hand, the TMB-low (TMB-L) group included, in addition to the rest of the pMMR-IHC/MSS-PCR patients, 6discordant cases: 5 dMMR-IHC/MSS-PCR and 1 pMMR-IHC/MSI-H-PCR. As described above, 4 of the 5 dMMR-IHC/MSS-PCR tumors were re-defined as pMMR following retesting/reviewing with IHC while the other one was likely to have been misdiagnosed considering its TMB load. All 6 cases were defined as MSS using NGS, and the notion that they have been misdiagnosed was further supported by their low TMB loads (1.1 ~ 20.2 mut/MB). Therefore, compared with conventional techniques, TMB estimation enabled by the NGS method may offer us more insights into the molecular features of CRC and identify an additional cohort of patient (MSS-TMB-H) who might benefit from immunotherapy. What’s more, for the the whole 430 CRC cohort, there are 9 cases with MSS and TMB higher than 29 muts/Mb, and 6 of them are POLE-mutated patient while the other 3 patients don’t harbor *POLE* or *POLD1*, indicating that there are other mechanism causing TMB-H in MSS group except *POLE* or *POLD1*. Additionally, the MSI-H or TMB-H (≥29 muts/Mb) CRC were both more common in the early stage tumors than in late stage tumors (MSI-H: stage I/II, 31.25%, III/IV, 68.75%, *p* = 0.006; TMB-H: stage I/II, 37.14%, III/IV, 62.86%, *p* = 0.056). (Table S3).

### TMB distribution and immune signature expression across subgroups in the Cancer genome atlas (TCGA) CRC dataset

**Fig. 5:**
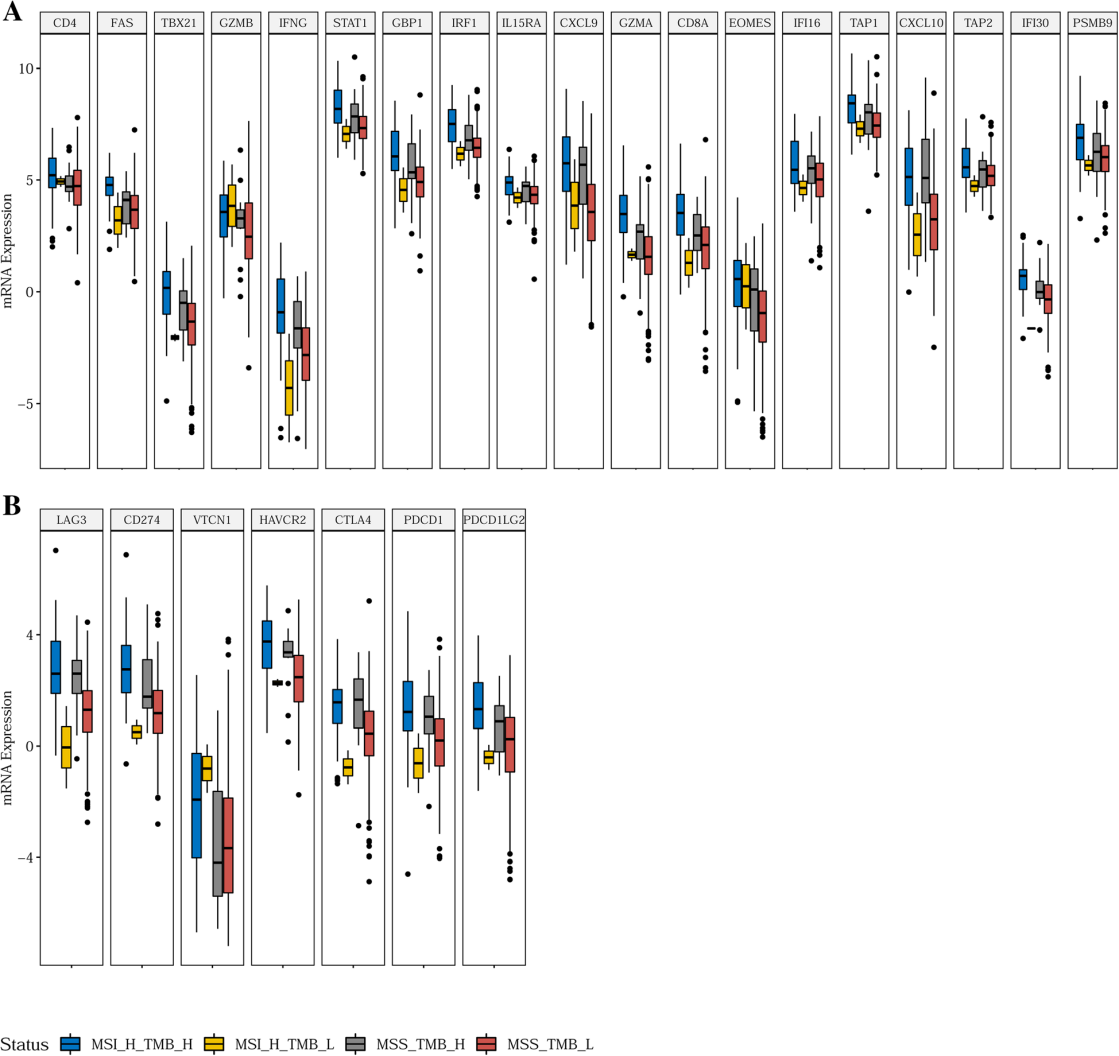
Expression profiles of immune signature genes in subgroups divided according to MSI (by PCR) and TMB (by NGS) statuses using RNAseq data of the TCGA CRC cohort. **a** Expression profiles of 19 immune activation-related genes; **b** Expression profiles of 7 immune checkpoint genes

Since it was revealed using our clinical cohort that some MSS patients may be TMB-H as a result of *POLE* mutations and this information could potentially expand the population of CRC who may benefit from ICIs, we sought to validate this notion by interrogating the MSI, TMB and RNAseq data of 533 CRC patients obtained from the TCGA database. Of the 533 tumors, 77 were MSI-H and 456 were MSS. As anticipated, the median TMB of the MSI-H group (24.5 muts/Mb) was significantly higher than that of the MSS tumors (2.2 muts/Mb) (*p* < 0.001) (Supplemental Figure 3). When dichotomized into TMB-H and TMB-L subgroups using a cut-off of 9 muts/Mb, as determined based on an optimal YOUDEN index of 0.94 with a sensitivity of 0.974 and a specificity of 0.967, following training using the TCGA cohort (Supplemental Figure 4), it was prominent that 15 tumors were MSS/TMB-H. Aberrations in *POLE* were detected in 46.7% (7/15) of the MSS-TMB-H, 1.3% (1/75) of the MSI-H/TMB-H, and 0.45% (2/441) of the MSS-TMB-L tumors respectively, further corroborating our hypothesis that *POLE* mutation is a major contributor to the discordance between MSI and TMB statuses. In order to better understand whether these subgroups differ in their ability to mount immune responses upon ICI treatment, the expression profiles of immune activation-related genes, immune checkpoint-related genes were compared among four subgroups: MSI-H/TMB-H, MSS-TMB-H, MSI-H/TMB-L, and MSS/TMB-L (Fig. 5a, b, Supplemental Table S2). Intriguingly, 15 of the 19 immune activation related genes had similar expression levels between the MSI-H/TMB-H and the MSS/TMB-H groups, leading to a 79% similarity. The 15 genes were CD4, CXCL10, CXCL9, EOMES, GBP1, GZMB, IFI16, IFNG, IL15RA, IRF1, PSMB9, STAT1, TAP1, TAP2, and TBX21. Conversely, among the 19 immune activation related genes 11 displayed significantly higher expression in the MSS-TMB-H group than in the MSS-TMB-L group (CD8A, CXCL10, CXCL9, GBP1, GZMA, IFI30, IFNG, IRF1, STAT1, TAP1, and TBX21), indicating that the two groups were different in their immune signatures. Among the 7 immune checkpoints examined, 6 (CD274, PDCD1, LAG3, PDCD1LG2, HAVCR2, and CTLA4) had similar expression in the MSI-H-TMB-H and the MSS-TMB-H groups, which was significantly higher than those of the MSS-TMB-L patients. In summary, 26 of the 26 (100%) immune signature genes were expressed at significantly higher levels in the MSI-H-TMB-H subset than in the MSS-TMB-L group, while the MSI-H-TMB-H and the MSS-TMB-H patients had similar profiles in 21 genes (81%). The MSS-TMB-H patients had significantly higher expression than the MSS-TMB-L group in 17 genes (65%). In short, the microenvironment of the MSS-TMB-H tumors was similar to that of the MSI-H-TMB-H tumors, but significantly more immune-responsive than that of the MSS-TMB-L tumors.

## Discussion

This work represents one of the first efforts to characterize the genomic landscape of Chinese CRC population on a large scale, and to inform CRC patient selection for ICI treatment according to MSI combined with TMB as determined using a single NGS panel. In a subset of 98 patients, our NGS method proved to be highly concordant with both PCR and IHC, especially PCR, in terms of MSI detection. Among the 7 cases of PCR-IHC inconsistency, 4 were confirmed to have been IHC-misdiagnosed and 1 was probably a PCR-misdiagnosis given its low TMB level, all of which were correctly defined using NGS, indicating that our NGS method was more accurate than conventional methods. Moreover, the advantages of NGS over PCR and IHC were also reflected in its ability to provide additional information regarding TMB levels and alterations in CRC-relevant genes to guide targeted therapy and immunotherapy and to inform hereditary cancer screening. TMB was also verified as a marker independent of MSI using both our clinical cohort and the TCGA CRC cohort. The high TMB loads of the MSS tumors in our cohort could possibly be solely attributed to the presence of *POLE* variations. Further immune signature analyses also revealed that MSI combined with TMB may improve identification of patients with higher immune responsiveness.

IHC and PCR are both widely utilized for MMR/MSI identification in clinical practice, however, they only correlated in 62.2% ~ 93% of cases according to previous reports, which may pose a challenge for selecting appropriate patients to receive ICI treatment [23–28]. For instance, in the CheckMate-142 study, a phase 2 trial evaluating nivolumab’s efficacy and safety in patients with dMMR/MSI-H metastatic CRC, 14 (19%) of the patients with locally determined dMMR/MSI-H tumors (10 assessed by IHC and 4 assessed by PCR) were centrally identified as MSS/MSI-L using PCR, indicating that discordance existed not only between local and central laboratories, but also between PCR and IHC, and this had important clinical implications as only 21% of the 14 patients had confirmed responses to nivolumab, remarkably lower than the 41% (16/39) response rate for the patients with consistent local-central and IHC-PCR results [5]. In our study, NGS combing MSI and TMB served as a reliable solution to such problems by providing not only accurate diagnosis itself but also supplemental evidence to validate the diagnosis from different angles.

In the subsequent analyses, it was observed in our series and the TCGA dataset that 1 in 16.5 (4/66) and 1 in 30.4(15/456) MSS patients presented with high TMB levels. Subgroup analyses of the expression profiles of immune signature genes further confirmed that TMB may dictate a tumor’s likelihood to benefit from ICIs regardless of its MSI status. These results are consistent with the trend observed in a recent publication by Goodman A.M. et al., where 1 in 18.4 (7972/1,446,624) MSS solid tumors was found to be TMB-H and the median progression-free survival for the MSS/TMB-H cases was significantly prolonged compared to that of the MSS/TMB-L/TMB-intermediate group (26.8 vs. 4.3 months, *P* = 0.0173), suggesting that the TMB information derived from NGS testing is clinically significant since it may expand the scope of ICI treatment to the MSS-TMB-H subpopulation which could otherwise be excluded [14]. At the same time, The CCTG CO.26 trial showed that in the subgroup with TMB ≥ 28, the advanced refractory MSS colorectal cancer patients treated with durvalumab plus tremelimumab had significantly better overall survival than the control arm, while for the subgroup with TMB < 28, the overall survival was similar between the two arms [29]. This result further proved MSS/TMB-H CRC could benefit from ICIs. Moreover, in the NICHE trial, which was the first neoadjuvant immunotherapy trial for CRC, 27% (4/15) of the pMMR tumors showed pathological response to ipilimumab and nivolumab, and the degree of CD8 + PD-1+ T cell infiltration was significantly higher in among the responders than in the non-responders in the pMMR group, indicating that microenvironment might be the underlying mechanism for MSS CRC benefiting from ICIs [30]. Currently, TMB was proved as an effective biomarker to predict the efficacy of nivolumab and ipilimumab in first line therapy for NSCLC (trial Checkmate 568), indicating that using NGS panel to evaluate TMB is promising in the clinical practice [31]. It is worth noting that whole exome sequencing (WES) was applied to evaluate TMB for the TCGA cohort, while an NGS panel was used for the clinical cohort. The panel selectively included 381 genes that are frequently mutated in tumor tissue, so the selection bias tended to result in a high TMB level. Therefore the panel-based TMB values could not be directly compared with the TMB values calculated by WES.

As for the mechanism underlying the MSS-TMB-H phenotype, the occurrence of spontaneous mutations in *POLE* and *POLD1*, which encodes the exonuclease (proofreading) domain of DNA polymerase epsilon and polymerase delta [20], respectively, may account for the high TMB in some cases. Multiple lines of evidence showed an association between *POLE* mutation and clinical benefit from ICIs [32–34]. Indeed, 100% (4/4) and 46.7% (7/15) of the MSS-TMB-H tumors in our clinical cohort and the TCGA cohort carried pathogenic mutations in *POLE*, which were substantially enriched compared to the 0% (0/94) and 1.9% (10/518) among the rest of the patients in our cohort and the TCGA cohort. Apart from the impact of defects in *POLE*, the high TMB loads of the other eight TCGA MSS-TMB-H tumors could potentially be explained by the presence of mutations in the DNA repair pathways [35].

Our study do have some limitations. It was retrospective in nature and the sample size of the clinical validation cohort was relatively small. The patients’ baseline characteristics such as tumor stage were also heterogeneous. By restricting clinical validation and TMB distribution analyses only to tumors having undergone testing by all three approaches (IHC, PCR and NGS), we might have introduced sampling bias. Indeed, up to 84.9% of the study population had advanced disease, but this was actually consistent with the main purpose of this study, which was to validate a novel diagnostic approach of advanced CRC. That being said, the applicability of the molecular profiling strategy described herein in early stage CRC patients awaits further investigation. Moreover, although the immune activation profiles of TCGA CRC cohort were employed as a surrogate to explore the differences in response to ICI treatment across different subgroups, they may not fully reflect the clinical efficacy of ICI treatment. Therefore, the notion that TMB combined with MSI status could further refine patient selection for ICIs warrants further investigation with a prospective study. In conclusion, our NGS panel-based method is both robust and tissue-efficient and enables comprehensive molecular diagnosis of CRC with special implications for ICI efficacy prediction.

## Conclusion

This study demonstrated that NGS panel-based method is both robust and tissue-efficient for comprehensive molecular diagnosis of CRC. It also underscores the importance of combining MSI and TMB information for identifying patients with a favorable immune microenvironment.

## Supplementary Information

**Additional file 1: Figure S1.** Number of somatic variants (TMB) detected in 430 Chinese CRC patients in the POLE mutation group and without POLE mutation group. **Figure S2.** The ROC curve of Chinese CRC cohort for the optimal cut-off point was in the upper-left area and was calculated based on the maximal Youden index. **Figure S3.** Number of somatic variants (TMB) detected in TCGA CRC patients in the POLE mutation group and without POLE mutation group. **Figure S4.** The ROC curve of TCGA CRC cohort for the optimal cut-off point was in the upper-left area and was calculated based on the maximal Youden index.

**Additional file 2: Table S1.** The Genes list of NGS panel MasterView.

**Additional file 3: Table S2.** The somatic and germline mutations information of the 430 CRC patients.

**Additional file 4: Table S3.** The molecular diagnostic result of 98 CRC patients.

**Additional file 5: Table S4.** The Expression profiles of immune activation-related genes, immune checkpoint-related genes compared among four MSI/TMB subgroups.

## Data Availability

The data used to support the findings of this study are available from the corresponding author upon request.

## Abbreviations

CRC: Colorectal cancer
MSI: Microsatellite instability
MMR: Mismatch repair
TMB: Tumor mutation burden
NGS: Next generation sequencing
IHC: Immunohistochemistry
PCR: Polymerase chain reaction
ICIs: Immune checkpoint inhibitors
LS: Lynch Syndrome
AUC: Area under the curve
ROC: Receiver operating characteristic
TCGA: The Cancer Genome Atlas
