# Supplementary figures and images for: A next-generation sequencing-based strategy combining microsatellite instability and tumor mutation burden for comprehensive molecular diagnosis of advanced colorectal cancer
Source: BMC Cancer. 2021 Mar 16;21:282. doi: 10.1186/s12885-021-07942-1 (PMC7962287; doi:10.1186/s12885-021-07942-1)

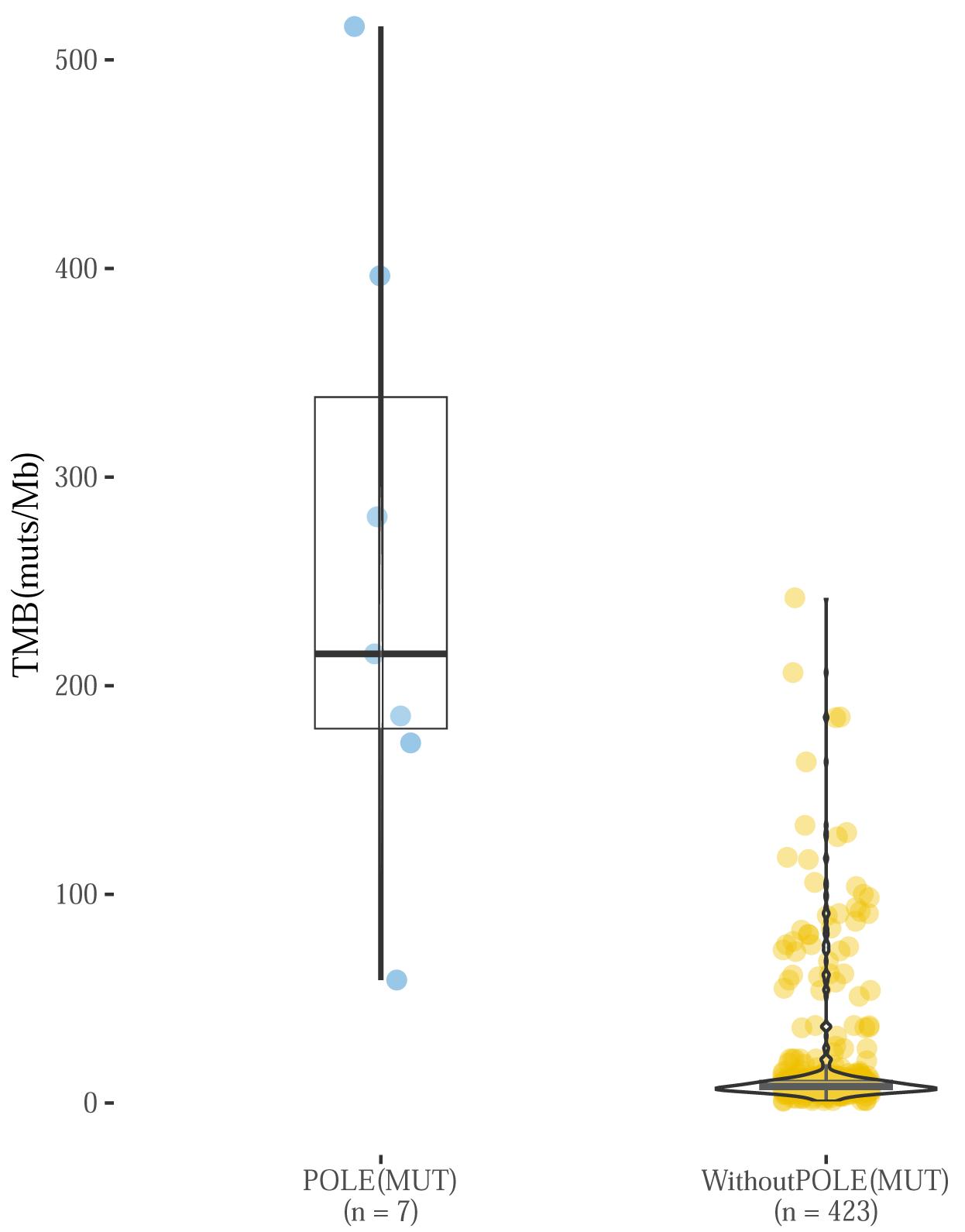

Supplement: Supplementary file 1 — Additional file 1: Figure S1. Number of somatic variants (TMB) detected in 430 Chinese CRC patients in the POLE mutation group and without POLE mutation group. Figure S2. The ROC curve of Chinese CRC cohort for the optimal cut-off point was in the upper-left area and was calculated based on the maximal Youden index. Figure S3. Number of somatic variants (TMB) detected in TCGA CRC patients in the POLE mutation group and without POLE mutation group. Figure S4. The ROC curve of TCGA CRC cohort for the optimal cut-off point was in the upper-left area and was calculated based on the maximal Youden index. [file 12885_2021_7942_MOESM1_ESM.zip › FigS1.tif]

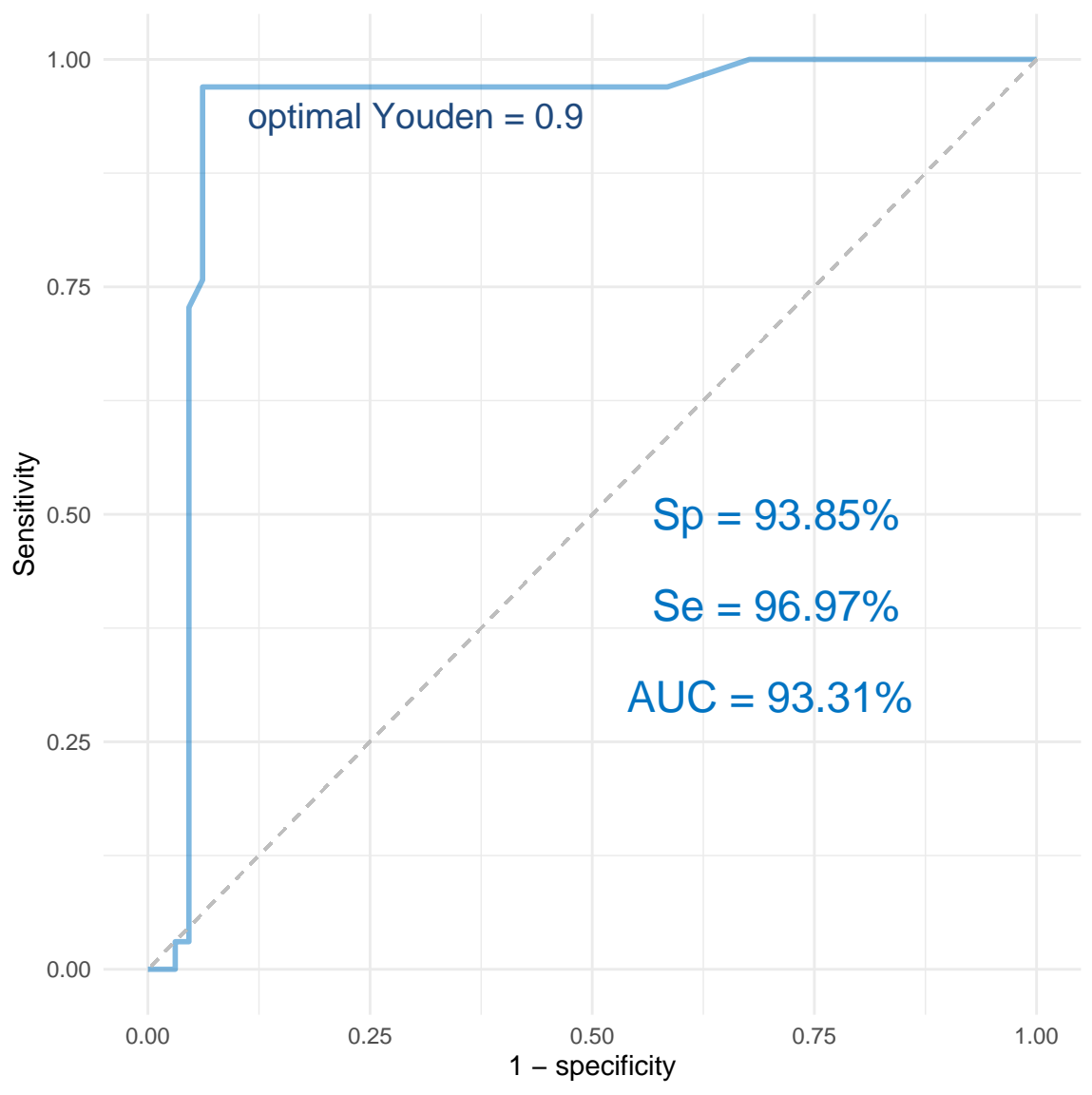

Supplement: Supplementary file 1 — Additional file 1: Figure S1. Number of somatic variants (TMB) detected in 430 Chinese CRC patients in the POLE mutation group and without POLE mutation group. Figure S2. The ROC curve of Chinese CRC cohort for the optimal cut-off point was in the upper-left area and was calculated based on the maximal Youden index. Figure S3. Number of somatic variants (TMB) detected in TCGA CRC patients in the POLE mutation group and without POLE mutation group. Figure S4. The ROC curve of TCGA CRC cohort for the optimal cut-off point was in the upper-left area and was calculated based on the maximal Youden index. [file 12885_2021_7942_MOESM1_ESM.zip › FigS2.pdf]

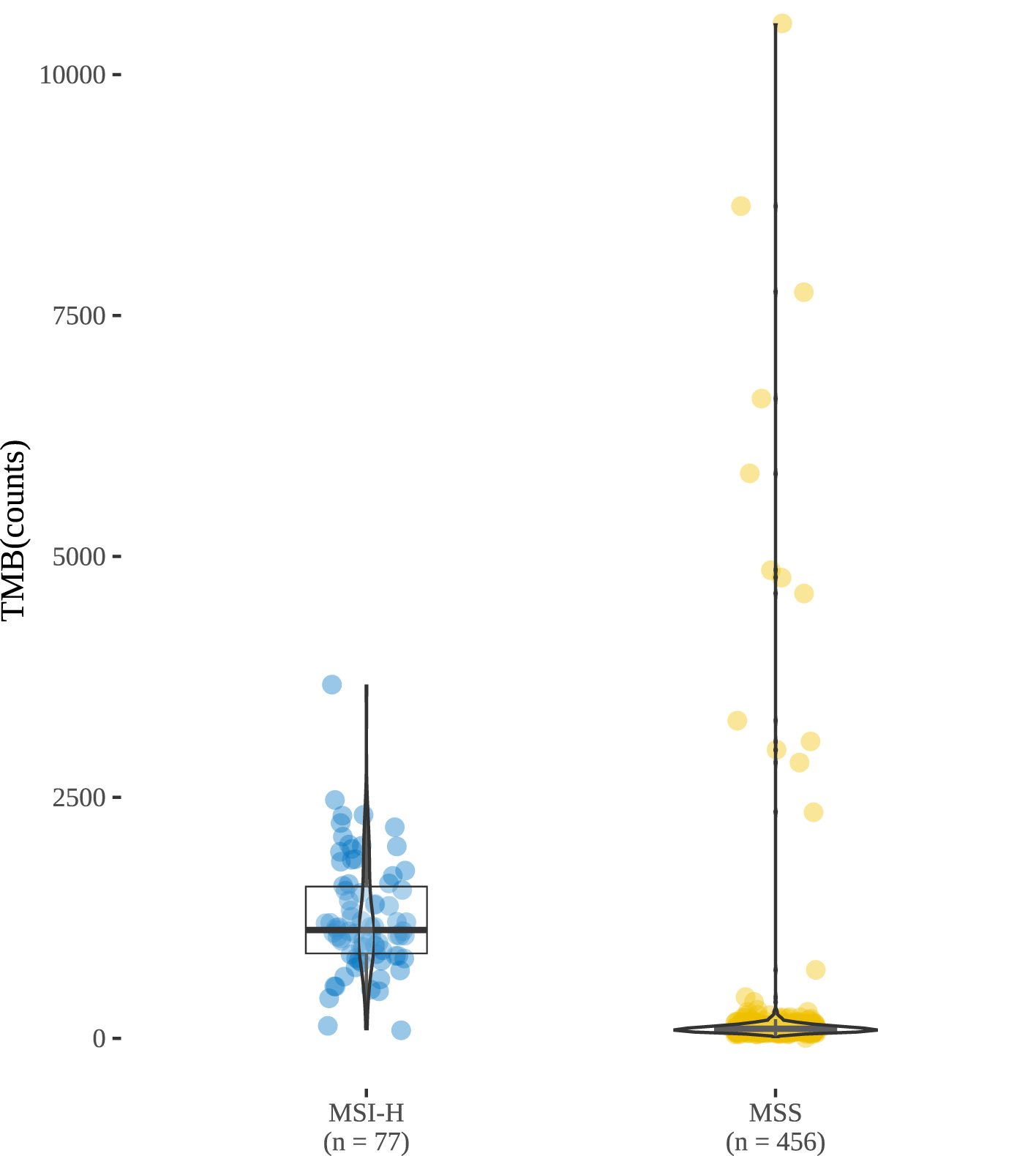

Supplement: Supplementary file 1 — Additional file 1: Figure S1. Number of somatic variants (TMB) detected in 430 Chinese CRC patients in the POLE mutation group and without POLE mutation group. Figure S2. The ROC curve of Chinese CRC cohort for the optimal cut-off point was in the upper-left area and was calculated based on the maximal Youden index. Figure S3. Number of somatic variants (TMB) detected in TCGA CRC patients in the POLE mutation group and without POLE mutation group. Figure S4. The ROC curve of TCGA CRC cohort for the optimal cut-off point was in the upper-left area and was calculated based on the maximal Youden index. [file 12885_2021_7942_MOESM1_ESM.zip › FigS3.tif]

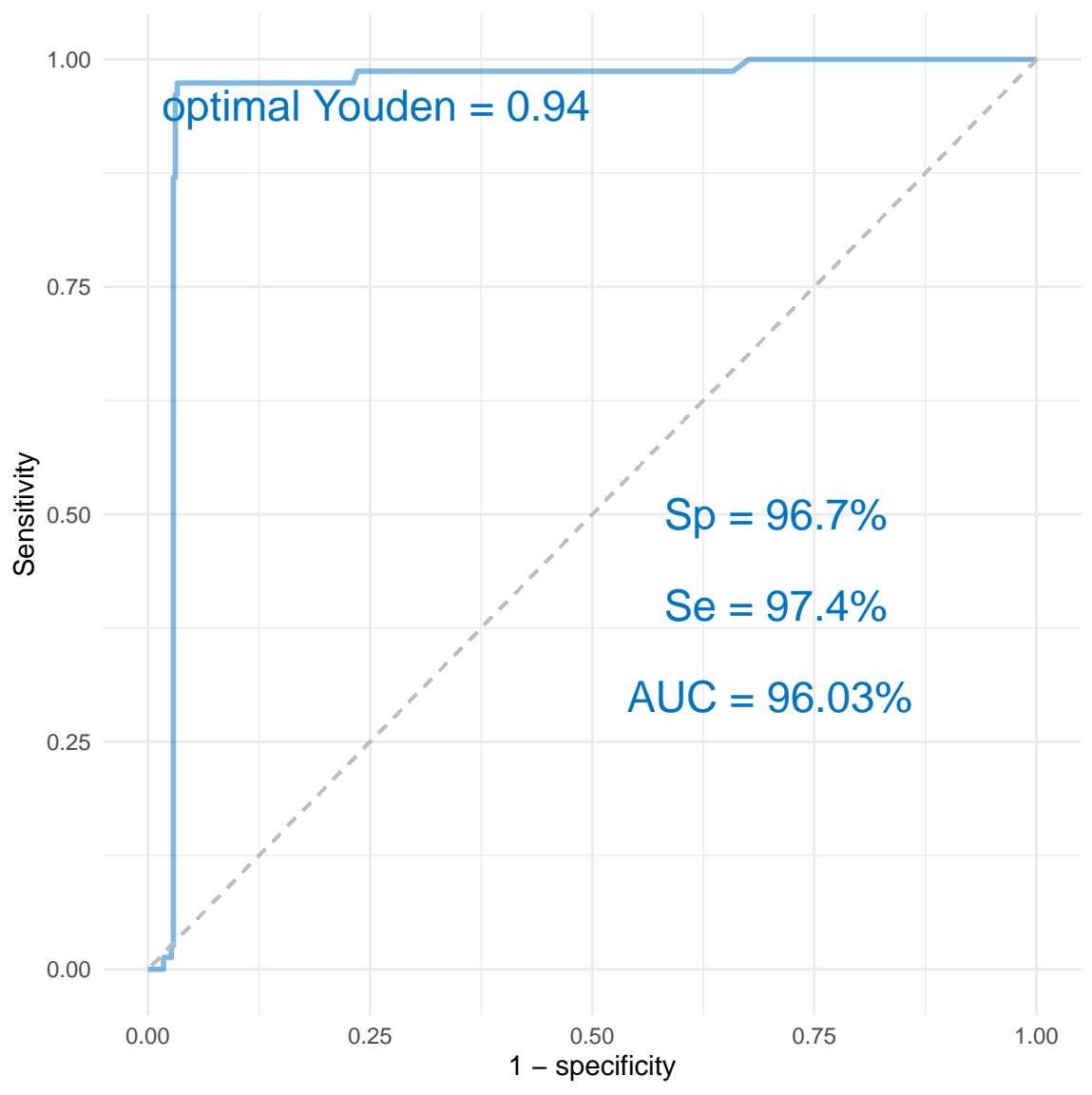

Supplement: Supplementary file 1 — Additional file 1: Figure S1. Number of somatic variants (TMB) detected in 430 Chinese CRC patients in the POLE mutation group and without POLE mutation group. Figure S2. The ROC curve of Chinese CRC cohort for the optimal cut-off point was in the upper-left area and was calculated based on the maximal Youden index. Figure S3. Number of somatic variants (TMB) detected in TCGA CRC patients in the POLE mutation group and without POLE mutation group. Figure S4. The ROC curve of TCGA CRC cohort for the optimal cut-off point was in the upper-left area and was calculated based on the maximal Youden index. [file 12885_2021_7942_MOESM1_ESM.zip › FigS4.pdf]
