# Supplementary material for: A next-generation sequencing-based strategy combining microsatellite instability and tumor mutation burden for comprehensive molecular diagnosis of advanced colorectal cancer
Source: BMC Cancer. 2021 Mar 16;21:282. doi: 10.1186/s12885-021-07942-1 (PMC7962287; doi:10.1186/s12885-021-07942-1)
Supplement: Supplementary file 2 — Additional file 2: Table S1. The Genes list of NGS panel MasterView. [file 12885_2021_7942_MOESM2_ESM.doc]

Table S1. Gene list for the 381 cancer gene panel.

ABL1 ABL2 ACVR1B ACVR2A ADAM29 ADGRA2 AKT1 AKT2 AKT3 ALK AMER1 APC AR ARAF ARFRP1 ARID1A ARID1B ARID2 ASXL1 ATM ATR ATRX AURKA AURKB AXIN1 AXL BAP1 BARD1 BCL2 BCL2L1 BIM BCL2L2 BCL6 BCOR BCORL1 BCR BIRC5 BLK BLM BMX BRAF BRCA1 BRCA2 BRD4 BRIP1 BTG1 BTK C11orf30 CARD11 CBFB CBL CCND1 CCND2 CCND3 CCNE1 CD274 CD79A CD79B CDC73 CDH1 CDK12 CDK4 CDK6 CDK8 CDKN1A CDKN1B CDKN2A CDKN2B CDKN2C CEBPA CHD2 CHD4 CHEK1 CHEK2 CIC CRBN CREBBP CRKL CRLF2 CSF1R CSK CSNK1A1 CTCF CTNNA1 CTNNB1 CUL3 CXCR4 CYLD CYP2C19 CYP2D6 DAXX DDR1 DDR2 DICER1 DNMT3A DOT1L DPYD EGF EGFR EP300 EPHA2 EPHA3 EPHA5 EPHA7 EPHB1 ERBB2 ERBB3 ERBB4 ERCC1 ERG ERRFI1 ESR1 ETV1 ETV4 ETV5 ETV6 EZH2 FAM135B FAM46C FANCA FANCC FANCD2 FANCE FANCF FANCG FANCL FAS FAT1 FBXW7 FGF10 FGF14 FGF19 FGF23 FGF3 FGF4 FGF6 FGFR1 FGFR2 FGFR3 FGFR4 FGR FH FLCN FLT1 FLT3 FLT4 FOXL2 FOXP1 FRS2 FUBP1 FYN GABRA6 GATA1 GATA2 GATA3 GATA4 GATA6 GID4 GLI1 GLI2 GLI3 GNA11 GNA13 GNAQ GNAS GRIN2A GRM3 GSK3B H3F3A HCK HGF HNF1A HRAS HSD3B1 HSP90AA1 IDH1 IDH2 IGF1R IGF2 IKBKE IKZF1 IL7R INHBA INPP4B IRF2 IRF4 IRS2 ITK JAK1 JAK2 JAK3 JUN KAT6A KDM5A KDM5C KDM6A KDR KEAP1 KEL KIT KLHL6 KMT2A KMT2C KMT2D KRAS LCK LIMK1 LMO1 LRP1 LRP1B LYN LZTR1 MAGI2 MAP2K1 MAP2K2 MAP2K4 MAP3K1 MAP4K5 MCL1 MDM2 MDM4 MED12 MEF2B MEN1 MET MITF MLH1 MPL MRE11A MS4A1 MSH2 MSH6 MST1R MTOR MUTYH MYB MYC MYCL MYCN MYD88 NEK11 NF1 NF2 NFE2L2 NFKBIA NKX2-1 NOTCH1 NOTCH2 NOTCH3 NPM1 NRAS NRG1 NRG3 NSD1 NTRK1 NTRK2 NTRK3 NUP93 PAK3 PALB2 PARK2 PAX5 PBRM1 PDCD1LG2 PDGFRA PDGFRB PDK1 PIK3C2B PIK3CA PIK3CB PIK3CD PIK3CG PIK3R1 PIK3R2 PKD2 PLA2G1B PLCG2 PMS2 POLD1 POLE PPP2R1A PRDM1 PREX2 PRKAR1A PRKCI PRKDC PRSS8 PTCH1 PTEN PTK2 PTK6 PTPN11 QKI RAC1 RAD50 RAD51 RAF1 RANBP2 RARA RB1 RBM10 RET RICTOR RIT1 RNF43 ROCK1 ROCK2 ROS1 RPTOR RUNX1 RUNX1T1 RXRA SDHA SDHB SDHC SDHD SETD2 SF3B1 SIK1 SLIT2 SMAD2 SMAD3 SMAD4 SMARCA2 SMARCA4 SMARCB1 SMO SNCAIP SOCS1 SOX10 SOX2 SOX9 SPEN SPOP SPTA1 SRC SRMS STAG2 STAT3 STAT4 STK11 STK24 SUFU SYK TAF1 TBX3 TCF7L2 TEK TERT TET2 TGFBR1 TGFBR2 TIE1 TMPRSS2 TNFAIP3 TNFRSF14 TNFSF11 TNK2 TOP1 TOP2A TP53 TPMT TSC1 TSC2 TSHR TYK2 U2AF1 UGT1A1 VEGFA VHL WEE1 WEE2 WISP3 WT1 XIAP XPO1 YES1 ZBTB2 ZNF217 ZNF703 ZNF750
